# Nijmegen breakage syndrome: case report and review of literature

**DOI:** 10.11604/pamj.2020.35.85.14746

**Published:** 2020-03-20

**Authors:** Brahim El Hasbaoui, Abdelhkim Elyajouri, Rachid Abilkassem, Aomar Agadr

**Affiliations:** 1 Department of Pediatrics, Military Teaching Hospital Mohammed V, Faculty of Medicine and Pharmacy, University Mohammed V, Rabat, Morocco

**Keywords:** Nijmegen breakage syndrome, chromosome instability, immunodeficiency, lymphoma

## Abstract

Nijmegen Breakage Syndrome (NBS) is a rare autosomalrecessive DNA repair disorder characterized by genomic instability andincreased risk of haematopoietic malignancies observed in morethan 40% of the patients by the time they are 20 years old. The underlying gene, NBS1, is located on human chromosome 8q21 and codes for a protein product termed nibrin, Nbs1 or p95. Over 90% of patients are homozygous for a founder mutation: a deletion of five base pairs which leads to a frame shift and protein truncation. Nibrin (NBN) plays an important role in the DNA damage response (DDR) and DNA repair. DDR is a crucial signalling pathway in apoptosis and senescence. Cardinal symptoms of Nijmegen breakage syndrome are characteristic: microcephaly, present at birth and progressive with age, dysmorphic facial features, mild growth retardation, mild-to-moderate intellectual disability, and, in females, hypergonadotropic hypogonadism. Combined cellular and humoral immunodeficiency with recurrent sino-pulmonary infections, a strong predisposition to develop malignancies (predominantly of lymphoid origin) and radiosensitivity are other integral manifestations of the syndrome. The diagnosis of NBS is initially based on clinical manifestations and is confirmed by genetic analysis. Prenatal molecular genetic diagnosis is possible if disease-causing mutations in both alleles of the NBN gene are known. No specific therapy is available for NBS; however, hematopoietic stem cell transplantation may be one option for some patients. Prognosis is generally poor due to the extremely high rate of malignancies. We present here a case of Nijmegen breakage syndrome associated with Hodgkin lymphomas and Combined variable immunodeficiency.

## Introduction

Nijmegen breakage syndrome is a rare autosomal recessive disease, belonging to a group of disorders often called chromosome instability syndromes, characterized by microcephaly at birth without neurological manifestations. Other important clinical features, more noticeable with age, include mild growth delay, premature ovarian insufficiency, predisposition to recurrent infections of various organs and a very high risk to develop malignancies in early age, most frequently of haematological origin. Combined immunodeficiency of both cellular and humoral response is an essential feature of the disease [1]. World-wide prevalence of NBS is estimated at 1:100,000 live births. However, NBS is particularly common in Eastern Europe with carrier frequencies as high as 1:155 in some populations. Chromosomal instability with characteristic rearrangements in peripheral T lymphocytes in the form of inversions and translocations involving chromosomes 7 and 14, and cellular sensitivity to ionising radiation (IR) in vitro are all characteristic for the disease and have diagnostic relevance. Identifying mutations in both alleles of the nibrin (NBN) gene (formerly NBS1) completes the diagnosis of NBS. Most NBS patients are of Slavonic origin and therefore, this frameshift mutation came to be called "Slavonic mutation". We present here a case of Nijmegen breakage syndrome associated with Hodgkin lymphomas and combined variable immunodeficiency.

## Patient and observation

**Figure 1:**
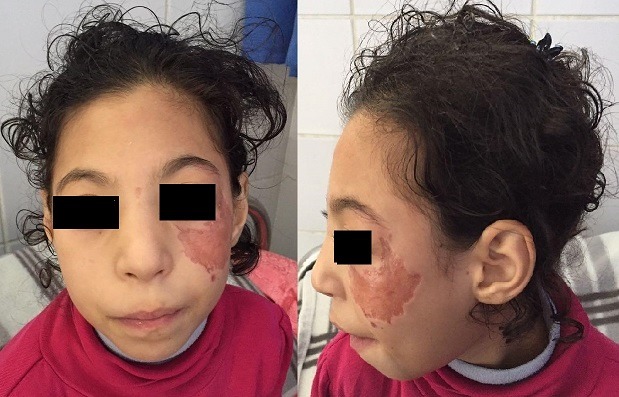
Facial features of our patient

**Figure 2:**
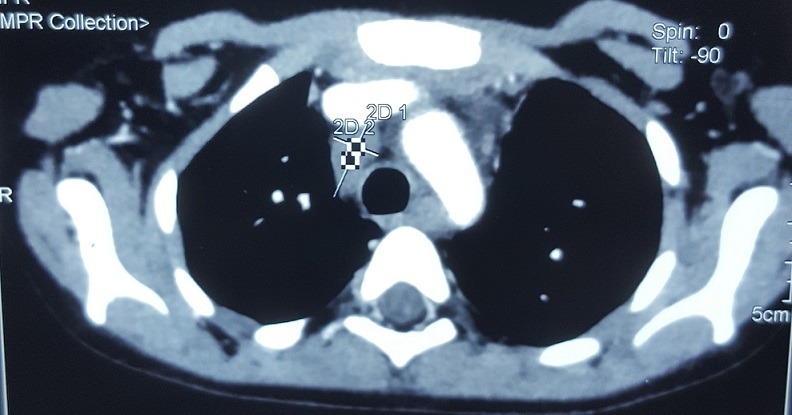
Initial radiologic exploration

We present here a case of Nijmegen breakage syndrome in an 8-year-old girl born of consanguineous parentage. Her birth histories, her family social history, and developmental milestones were unremarkable. She was born at a local birthing centre, at full term with no antenatal or perinatal complications, attended solely by midwives, she was exclusively breast-fed, food diversification was started at 6 months old, her weight, length and psychomotor development were within the normal range, the child was described as a good eater on a normal diet, and was thriving appropriately. Furthermore, the girl presented recurrent episodes of respiratory tract infections such as pneumonia and sinusitis since 2 years of age, indeed there was history of 5 hospitalizations at a health centre; the last one goes back to the age of 6, then she was sent back to our department for a complete management. On admission, our patient had characteristic facial appearance with a combination of receding forehead, receding mandible and prominent midface, she had also epicanthal folds, large ears, and sparse. In addition, profound microcephaly was noticed (-3SD) that contrasted with normal developmental milestones and good comprehension, while no motor problems and no delayed speech were observed. On the other side, mild growth delay was observed (-2SD on the weight and length) with periorbital squamous lesions (Figure 1). On the basis of this clinical presentation, a tentative diagnosis of Nijmegen Breakage Syndrome had been made; it was confirmed later by genetic analysis. Because of recurrent episodes of respiratory tract infections and susceptibility to develop an immune deficiency further laboratory work-up was notable for increased Erythrocyte Sedimentation Rate (ESR) of 96 mm/h, and combined variable immunodeficiency (lymphocytopenia, T helper cell and B cell count significantly decreased, IGA and IgG4 deficiency). In accordance to this diagnosis and the fact that patients with NBS have a high risk for developing malignancy, predominantly Hodgkin lymphomas (HL) and non-Hodgkin lymphomas (NHL) the major cause of death in these individuals, initial radiological exploration showed some multiple lymph nodes (mediastinal, hilar and supraclavicular). Moreover, there was a small mass in the left lung with the splenic nodule Figure 2. Biopsy of lymph nodes, pulmonary and splenic nodules had established the diagnosis of nodular sclerosing classical HL while histological examination of the bone marrow showed no infiltration by HL. These findings are consistent with Hodgkin lymphomas Ann Arbor stage IVB. The initial step of management was based on systemic chemotherapy in combination with gamma-globulin replacement therapy every week. Overall, we applied eight cycles of chemotherapy, two of them COP two OEPA, and we were able to escalate full doses of COPDac, in spite of decrease of white blood count that was managed by using granulocyte stimulating factor (G-CSF). Follow-up MRI was performed at 3, 6, 12 and 18-months after initial diagnosis and continued to show complete remission. At 20-months after the initial diagnosis, the patient does not exhibit any clinical sequelae of the disease.

## Discussion

In 1981, Weemaes *et al*. described two brothers with congenital microcephaly, immunodeficiency, recurrent respiratory infections, and chromosomal breakage. This new disorder is called Nijmegen breakage syndrome (NBS) [2]. In 1985, Seemanova *et al*. described a high risk for lymphoreticular malignancy in nine NBS patients [3]. Until 1998 the diagnosis of NBS was possible only on clinical ground and at the cytogenetic level. In 1998, Varon *et al.* demonstrated that nearly all NBS patients are homozygous for the same founder mutation deletion of 5 bp (657del5) in the NBS1 gene, encoding the protein nibrin [4]. The gene underlying NBS is NBN whose product, nibrin (p95), that is part of a trimeric complex together with MRE11 and RAD50 (the MRN complex). This complex is evolutionarily highly conserved, and consists of the proteins Mre11, Rad50 and Xrs2, whereby Xrs2 represents the functional orthologue of nibrin. The MRN complex is involved in DNA double strand break (DSB) repair by both homologous recombination and nonhomologous end joining, the two principle DSB repair pathways of mammalian cells. Nibrin is apparently a sensor of DSBs as it is required for relocation of the complex to the sites of DSBs after irradiation [5]. In addition to a direct role in DNA repair, the complex is also involved in the activation of ATM, the protein mutated in Ataxia telangiectasia, and thus in the triggering of cell cycle checkpoints [6]. The vast majority of NBS patients are homozygous for a founder mutation, c.657_661del5 (p.K219fsX19), in exon 6 of the gene [4]. Alternative translation of the NBN mRNA leads to a carboxyterminal fragment, p70-nibrin, with residual function [7]. Nijmegen breakage syndrome is a rare disease and there are no reliable estimates of its prevalence. The number of known patients identified worldwide increased significantly when the disease-causing gene, NBN, was identified. Apart from over 150 subjects reported in the medical literature [8]. The disease seems to occur worldwide, but with a distinctly higher prevalence among Central European and Eastern European populations, i.e. in the Czech Republic, Poland, Russia and Ukraine [9,10]. The proportion of patients identified in these populations correlates with a high carrier frequency of the major NBN mutation, c.657_661del5 (p.K219fsX19), estimated to be 1 case per 177 new-borns, clearly the result of a founder effect [11]. The clinical phenotype of NBS consists of several cardinal features, such as progressive microcephaly, which influences facial phenotype, mild growth delay, premature ovarian failure, cellular and humoral immunodeficiency predisposing to recurrent infections, and an exceptionally high risk of cancer development at an early age. A hallmark symptom of NBS is microcephaly, which is observed from birth onwards and should alert a neonatologist or a paediatrician, [12,13]. Microcephaly in NBS is progressive and can be as severe as in older children contrasting with normal developmental milestones in the majority of NBS children [13]. It is striking to observe an infant or a toddler with severe microcephaly but with no motor problems and with very good comprehension.

On the other side, delayed speech development is common, and speech therapy is needed to correct articulation problems. Intelligence was shown to vary from normal to mild or moderate mental retardation. The dysmorphic facial features are very similar among all patients with NBS and become more obvious with age. In fact, the prominent midface is emphasized by the sloping forehead and the receding mandible, which seems to be secondary to the underdevelopment of the cranium. Other facial characteristics are subtler and diverse as palpebral fissures are upwardly slanted in most patients, and the shape of the nose may be both long and beaked as well as upturned with anteverted nostrils. In individual patients, cleft lip/palate or choanal atresia have also been described. NBS patients are prone to various infections, sinusitis, pneumonia and/or bronchopneumonia that result in bronchiectasis in some patients, are particularly frequent with, in some cases, a fatal outcome. Agammaglobulinemia has been reported for about one third of NBS patients whilst in others the humoral immune deficiency is more variable, deficiencies of IgA or IgG4, alone or in combination are, however, common. A minority of patients, about 10%, have normal Ig status [14]. Cellular immunity is more consistently deficient in NBS patients. T-lymphocyte proliferation in response to mitogenic stimuli is reduced in more than 90% of patients. Whilst CD8+ cells are generally within the normal range, reduced proportions of CD3+ and CD4+ T cells are found in most patients. Consequently, the CD4+/CD8+ ratio is reduced. Mild to moderate lymphopenia has also been reported and in a study of such patients a failure of T cell regeneration in the thymus was postulated to be compensated for by non-thymic pathways [15]. Moreover, patients with NBS have a high risk for developing malignancy, the major cause of death in these individuals. Of all the chromosomal instability syndromes, the incidence of cancer in NBS patients is one of the highest. By the age of 20 years, over 40% of NBS patients develop a malignant disease, predominantly of lymphoid origin. Non-Hodgkin lymphomas (NHL) of B and T cells are the most common, predominantly diffuse large B cell lymphoma (DLBCL), and T-cell lymphoblastic lymphoma (TLBL), however, Burkitt and Burkitt-like lymphomas are also encountered [16]. Importantly, NBS patients are also sensitive to ionising irradiation (IR) and its therapeutic use in undiagnosed patients can be fatal [17]. This radiosensitivity is, of course, reflected by an at least two-fold increase in cell death after an IR treatment of primary or immortalised NBS patient cells. IR produces DNA DSBs as its most serious lesion and NBS cells are sensitive to many other mutagens which produce DSBs or which make lesions repaired via DSBs as an intermediate: bleomycin, streptonigrin, etoposide, camptothecin, and the cross-linking agent mitomycin C [18]. In addition to clinical and cellular hypersensitivity to DSB-inducing agents, NBS is characterised by increased spontaneous and IR-induced chromosome breakage. On the other side, growth retardation is a cardinal symptom of NBS. Although birth weight and size are typically within the normal range, short stature is usually apparent by 2 years of age. Body weight remains proportional to body length. A reduced proliferative capacity of patient cells could be inferred from this symptom and is supported by several studies. Other anomalies can be described in NBS patients such as skeletal, skin and hair, genito-urinary system abnormalities.

The diagnosis of NBS is initially based on clinical manifestations and is confirmed by genetic analysis. Cytogenetics and molecular genetics can be utilized for the final diagnosis. Cytogenetic analysis of standard PHA-stimulated peripheral blood lymphocytes (T cells) allows detection of spontaneous chromosome instability; however, this may be hampered by the poor response of NBS patient lymphocytes to mitogens [19]. The constitutional karyotype is generally normal; however, a broad spectrum of abnormalities can be observed in 10-60% of cells. Among the aberrations most commonly found are open chromatid and chromosome breaks, aneuploidies, marker chromosomes, partial endo-reduplication and structural rearrangements [20]. Inversions and translocations, involving two different loci in chromosomes 7 and 14 are particularly characteristic for NBS and are found in the vast majority of cases. Significantly, the most frequently observed breakpoints are located in chromosome bands 7p13, 7q35, 14q11, and 14q32. There is no specific therapy available for NBS. Due to the specific basic defect underlying immunodeficiency and sensitivity to IR, patients with NBS require multidisciplinary medical management and long-term follow-up. As in other combined immunodeficiency syndromes, NBS patients generally need gamma-globulin replacement therapy due to IgG deficiency. The overall consensus among clinical immunologists is that an IVIG or SCIG dose that maintains a serum IgG level over 5.0 g/l (at least 4-6 g/l) is desirable. It is recommended that NBS patients should not be vaccinated with live bacterial or viral vaccines. In case of NBS patients diagnosed with lymphoproliferative disorders receive modified dosages of chemotherapy due to reduced treatment tolerance and high risk of life-threatening infectious complications. However, any defined modifications of standard protocols have been designed for patients treated due to leukemia or lymphoma in course of chromosomal instability disorders. It is common practice to modify treatment for NBS patients by limiting the doses of some chemotherapy agents (cyclophosphamide, ifosfamide) and even avoiding others (epipodophyllotoxins). The intensity of therapy is usually adapted to individual risk factors and tolerance [21]. Primary immunodeficiency diseases and lymphoid malignancy observed in NBS characterized by increased chromosomal instability or radiosensitivity pose a significant therapeutic challenge when bone marrow transplantation is considered. Hematopoietic stem cell transplantation (HSCT) has traditionally not been attempted in NBS because of concerns about excessive chemo- or radiotherapy induced toxicity. Additionally, transplant related toxicity such as graft versus host disease and the secondary immunodeficiency that accompanies it can further increase the risk for secondary malignancy. Early, etiologically correct diagnosis is crucial for appropriate preventive care with consequent protection against ionizing irradiation and with reduced chemotherapy of malignancy.

## Conclusion

Nijmegen breakage syndrome diagnosis is based on clinical features including microcephaly with no or very mild psychomotor and neurological lesions, recurrent infections and occurrence of lymphoid malignancies. Patients presenting these clinical characteristics should be subjected for genetic evaluation, which includes family history, cytogenetic abnormalities involving mostly two loci on chromosomes 7 (TCR genes cluster) and 14 (immunoglobulin heavy chain gene cluster) and search for biallelic mutations in NBN gene with the specific focus on c.657_661del5. No specific treatment is available for NBS patients beyond γ-globulin replacement therapy. Indeed, treatment of malignancy in NBS is particularly difficult since the underlying gene, nibrin (NBN), is involved in the cellular response to DNA damage. The consequent hypersensitivity of NBS patients severely limits therapeutic options and standard chemotherapeutics such as cyclophosphamide must be used at lower doses and radiotherapy completely avoided.

## Competing interests

The authors declare no competing interests.
